# Antibiotic Exposure and Risk of Allograft Rejection and Survival After Liver Transplant: An Observational Cohort Study From a Tertiary Referral Centre

**DOI:** 10.1111/tid.70026

**Published:** 2025-03-28

**Authors:** Olivia C. Smibert, Sara Vogrin, Marie Sinclair, Avik Majumdar, Mohamed Nasra, Dinesh Pandey, Hossein Jahanabadi, Jason A. Trubiano, Kate A. Markey, Monica A. Slavin, Adam Testro, Jason C. Kwong

**Affiliations:** ^1^ Sir Peter MacCallum Department of Oncology University of Melbourne Parkville Victoria Australia; ^2^ Department of Infectious Diseases Peter MacCallum Cancer Centre Melbourne Victoria Australia; ^3^ National Centre for Infections in Cancer Peter MacCallum Cancer Centre Melbourne Victoria Australia; ^4^ Department of Infectious Diseases and Immunology Austin Health Melbourne Victoria Australia; ^5^ Department of Infectious Diseases University of Melbourne The Peter Doherty Institute for Infection and Immunity Melbourne Victoria Australia; ^6^ Department of Medicine St Vincent's Hospital Victoria Parade University of Melbourne Fitzroy Australia; ^7^ Liver Transplant Unit Austin Health Melbourne Victoria Australia; ^8^ School of Medicine Dentistry and Health Sciences the University of Melbourne Melbourne Victoria Australia; ^9^ Data Analytics Research and Evaluation (DARE) Centre The University of Melbourne and Austin Hospital Melbourne Victoria Australia; ^10^ Clinical Analytics and Reporting Performance Reporting and Decision Support Austin Health Melbourne Victoria Australia; ^11^ Translational Science and Therapeutics Division Fred Hutchinson Cancer Center (FHCC) Seattle Washington USA; ^12^ Department of Medicine University of Washington Seattle Washington USA; ^13^ Department of Medicine Memorial Sloan Kettering Cancer Center New York New York USA; ^14^ Weill Cornell Medical College New York New York USA; ^15^ Department of Microbiology & Immunology The Peter Doherty Institute for Infection and Immunity the University of Melbourne Melbourne Victoria Australia

**Keywords:** antibiotics, liver transplant, microbiota, rejection, survival

## Abstract

**Introduction:**

Our goal is to understand whether there is an association between Abx exposure—and the inferred downstream damage to the intestinal microbiome—and the key patient outcomes of overall survival and rejection following liver transplant.

**Methods:**

We conducted a retrospective cohort study of 462 liver transplant recipients treated at a multistate liver transplant (LTx) service during a 7‐year period. The association between antibiotic exposure and outcome was tested across models that addressed antibiotic spectrum, duration, and timing relative to transplant. Cox proportional hazard regression was used to evaluate the relationship between antibiotics with survival and rejection.

**Results:**

The observed 1‐year survival in this cohort was 95% (95% CI: 93%, 97%), and 20.8% of patients (96/462) experienced rejection at 1 year. In multivariable analyses, exposure to anaerobe‐targeting antibiotics for longer than 14 days pretransplant (*p* = 0.055) or posttransplant (*p* = 0.040) was significantly associated with reduced 1‐year survival. In multivariable analyses, exposure to any anaerobe‐targeting Abx posttransplant was significantly associated with an increased risk of rejection (*p* = 0.001).

**Conclusions:**

Exposure to anaerobic spectrum antibiotics either before or after LTx was associated with poor outcomes during the first year posttransplant and provides an impetus to further characterize the relationship between antibiotic use, microbiota disruption, and cellular immunity in liver transplantation.

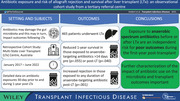

## Graphical abstract

Because the relationship between the gut microbiota and liver transplant outcomes is not well described, it is possible that there are, as yet, unrecognized negative impacts of antibiotic exposures on key liver transplant‐related outcomes. We observed a negative association between antibiotic exposures, particularly those with an anaerobic spectrum of activity, and post‐liver transplant outcomes

**Abbreviations**
Abx: Antibiotics
CAR T: chimeric antigen receptor T cell therapy
EMR: electronic medical record
ESLD: end‐stage liver disease
LTx: liver transplant
MELD: model for end‐stage liver disease
OS: overall survival
PFS: progression‐free survival

## 1 Introduction

Bacterial infections are a frequent and potentially life‐threatening complication for patients with end‐stage liver disease (ESLD) and may compromise suitability for transplant [1, 2, 3]. Infection is also the single greatest cause of mortality during the first few months following liver transplant (LTx) [4, 5], estimated to occur in upwards of 50% of recipients. While antibiotics are a critical aspect of care, they are also linked with *Clostridioides difficile* colitis and colonization with multidrug‐resistant bacteria [6, 7, 8, 9]. These effects are thought to be mediated through disruption of the healthy gut microbiota. Because the relationship between the gut microbiota and LTx outcomes is not well described, it is possible that there are, as yet, unrecognized negative impacts of antibiotic exposures on key LTx‐related outcomes. We hypothesized that exposure to antibiotics during the peri‐transplant period might be associated with the risk of rejection and survival during the first year following LTx. To test this hypothesis, we performed a retrospective cohort study among recipients of LTx at an Australian multistate transplant service to determine whether the duration, timing, and spectrum of antibiotic activity administered during the period immediately prior to and following transplant is independently associated with 1‐year survival and risk of rejection.

## 2 Methods

### 2.1 Study Design

A retrospective cohort study of all patients who received an LTx at a single tertiary health center in Melbourne, Australia, was conducted. Austin Health performs all LTxs for the two Australian states of Tasmania and Victoria. Patients were identified, and data were extracted from the Austin Health Liver Transplant database and health service electronic medical record (EMR). The study was approved by the local ethics committee HREC/91050/Austin‐2022.

### 2.2 Study Population, Data Collection, and Definitions

Patients were included if they were > 18 years old and underwent LTx between January 2015 and June 2022. Recurrent transplants during the study period were excluded. All transplant recipients are followed up and managed long‐term at the primary transplant site. Details of any complications or admissions occurring at peripheral healthcare sites are manually uploaded and documented in the institutional transplant database. Indications for transplantation and definitions of patient and donor characteristics are provided in Supporting Information. Surgical complications after transplant were categorized into the following: biliary anastomotic site, thrombosis of the hepatic artery or portal vein, intra‐abdominal hematoma, or other. Management of surgical complications was defined as surgical with any return to the operating room, radiological when managed via interventional radiology and without a surgical approach, or with medical‐only management when neither surgical nor radiological intervention was undertaken. Bloodstream infection (BSI) after transplant was defined as any positive blood culture identified via EMR considered to be clinically significant based on the administration of directed antibiotic treatment. All available determinations of tacrolimus trough concentrations undertaken during the first year following transplants were extracted from the health service EMR for each patient. Tacrolimus levels undertaken at remote pathology services are undertaken on the same testing platform and are routinely imported into the institutional transplant database and were also extracted. Rejection episodes were identified from the EMR using the following ICD‐10 codes: T86.4 or T86.88 and cross‐referenced with the liver biopsy histopathology from the EMR. Histopathological diagnoses of rejection were made based on the results of liver biopsy with grading based on the Banff schema rejection activity index (RAI) [10, 11].

### 2.3 Centre‐Specific Protocols

#### 2.3.1 Immunosuppression

The local protocol for transplant immunosuppression consists of initial triple therapy commenced immediately after the transplant. This regimen consists of steroids tapered over the first month posttransplant, mycophenolate mofetil initiated perioperatively, and either basiliximab on Day 1 postoperatively or tacrolimus. Tacrolimus is started to maintain trough levels between 7–10 ng/mL for the first 3 months and then subsequently reduced to between 6–8 ng/mL thereafter with adjustment of tacrolimus dosing at the discretion of the senior transplant clinician.

#### 2.3.2 Antibiotic Exposures

Local protocols for primary and secondary prophylaxis of spontaneous bacterial peritonitis are with trimethoprim + sulfamethoxazole 160 + 180 or ciprofloxacin as a second line. Routine peri‐transplant antibiotic therapy consists of piperacillin + tazobactam 4.5 g for three doses. Local guidelines for empiric treatment of posttransplant complications, including bile leak, cholangitis, septicemia, and return to the theater, are for piperacillin + tazobactam 4.5 g. There is no protocolized empiric carbapenem use unless in the presence of a microbiologically confirmed infection. Teicoplanin for three doses posttransplant is routine when the patient is documented to be Van B VRE colonized pretransplant. For patients transplanted in fulminant hepatic failure, routine pretransplant antibiotics consist of piperacillin + tazobactam 4.5 g as a bridge to transplant. Posttransplant antibiotic prophylaxis with trimethoprim + sulfamethoxazole for 6 months and antiviral prophylaxis with valganciclovir for high‐risk (CMV D+/R−) or valaciclovir (for all other patients) is undertaken for a minimum of 6 weeks and at the discretion of the treating clinician.

#### 2.3.3 Study Outcomes

The primary outcomes were all‐cause mortality and incidence of first biopsy‐proven rejection during the first 1 year posttransplant.

#### 2.3.4 Primary Exposure Variable

The primary exposure variable was an antibiotic spectrum of activity for all antibiotics prescribed during the 30 days prior to transplant and 1 year following, both inpatient and outpatient prescribed. The antibiotic spectrum was classified as being either broadly active against anaerobic organisms of the gastrointestinal tract or not [12]. The following antibiotics were classified as having anaerobic spectrum activity: piperacillin–tazobactam, ticarcillin–clavulanate, meropenem, imipenem–cilastatin, amoxicillin‐clavulanic acid, moxifloxacin, clindamycin, and metronidazole. Antibiotic therapy was measured by cumulative days of exposure. Patients were considered to have received a day of antibiotic exposure if they received at least a single dose of a given antibiotic on a given calendar day via any route of administration. Days where multiple different antibiotics were administered were counted cumulatively; that is, a day on which two different antibiotics were administered was counted as 2 days of exposure. Antibiotic exposure was divided into one of the following time periods: the 30 days preceding transplant or during the 1 year after transplant. Further details regarding the primary exposure variable are provided in the Supporting Information.

## 3 Statistics

The association between antibiotic exposure and outcome was tested across models that addressed the antibiotic spectrum of activity and duration of exposure. To assess the impact of the timing of antibiotic exposure relative to transplant, separate models for before and after transplant antibiotic exposures were undertaken. Posttransplant antibiotic exposure was entered as a time‐varying covariate to ensure that only antibiotic exposure prior to the outcome of interest was included in the model. Additional statistical methods and a summary of models undertaken are presented in the Supporting Information and Table S1. The primary analyses excluded routine prophylactic antibiotics, including per‐transplant routine perioperative antibiotics, which were administered to 100% of the cohort. Sensitivity analyses were undertaken on the subgroup of patients who received tacrolimus immunosuppression (438/462) to explore the association between antibiotic exposure and outcomes. Survival curves are displayed using the Kaplan–Meier method. Cox proportional hazard regression was used to evaluate the relationship between demographic characteristics and antibiotic exposure with survival and rejection. To address the potential risk of confounding by antibiotic indication for the posttransplant analyses, surgical complications and BSI were also included as time‐varying covariates. Other confounders were defined as variables with *p* < 0.10 on univariable analysis and were included in the multivariable models. Results are presented as hazard ratios (HR, for survival). For all analyses, two‐sided alpha < 0.05 was considered statistically significant. Analyses were performed using STATA 18 (StataCorp LLC, College Station, TX).

## 4 Results

### 4.1 Demographics and Clinical Characteristics

A total of 492 LTxs were performed during the study period. Thirty patients underwent a second LTx with a median of 18 days (IQR 3–288 days) following the first, leaving 462 recipients for the primary analyses. A summary of recipient and donor characteristics is presented in Tables 1 and S2. Surgical complications occurred in 8.9% (41/462) a median of 2 days (IQR; 1, 7) after transplant, with 78.0% (32/41) occurring within the first 30 days and the majority managed operatively (80.1%). Posttransplant outcomes are presented in Table S3. No association was observed between posttransplant surgical complications and either 1‐year OS or rejection (Tables S4 and S5). A total of 48 transplant recipients (10.4%) experienced a BSI following transplant at median Day 5 (IQR; 1, 21). There was a significant association observed between posttransplant BSI and reduced 1‐year OS (HR 2.24; 95% CI 1.19–4.19; *p* = 0.012) but no association between BSI and rejection (Tables S4 and S5). Approximately 5% (24/462) of recipients died during the first year posttransplant, and 13.6% (63/462) died during follow‐up, with a median time to death of 495 days (IQR 110, 1144). Infection was the primary cause of death in one instance and listed as a contributing cause in five others. Allograft rejection occurred in 22.1% (102/462) after a median of 15 days posttransplant (IQR: 9–53), and with 79.4% (81/102) occurring by Day 90. A total of 24,615 blood samples were analyzed for tacrolimus trough concentrations from 438/462 (95.0%) of participants, corresponding to a median number of 47 ± 24 trough levels per patient per year analyzed.

**TABLE 1:** Clinical characteristics of 462 liver transplant recipients.

Baseline characteristics	*N* = 462
Age, median (IQR)	56 (47, 62) (*n* = 462)
Sex (male)	294 (63.6%)
BMI, median (IQR)	27 (24, 32) (*n* = 436)
BMI categories	
<25	165 (35.7%)
25–30	127 (27.5%)
>30	144 (31.2%)
CCI categories	
0–2	67 (14.5%)
3–5	175 (37.9%)
>6	213 (46.1%)
Missing data	7 (1.5%)
Indication	
Autoimmune	29 (6.3%)
Biliary	55 (11.9%)
Alcohol	87 (18.8%)
Hepatitis B	44 (9.5%)
Hepatitis C	101 (21.9%)
NASH	53 (11.5%)
Redo	18 (3.9%)
Other	75 (16.2)
Concurrent HCC	112 (24.5%)
MELD categories	
<10	70 (15.2%)
11–19	94 (20.3%)
20–30	106 (22.9%)
>31	192 (41.6%)
VRE colonized	143 (31.0%)
MDRO gram‐negative colonized	65 (14.1%)
Medications	
Proton pump inhibitors	163 (35.3%)
Lactulose	173 (37.4%)
Rifaxamin	150 (32.5%)
Transplant waitlist days, median (IQR)	99 (23, 287)
Utilization of healthcare during 30 days prior to transplant	
Hospital admission	339 (73.4%)
ICU admission	65 (14.1%)
Hemodialysis	43 (9.5%)
Donor category	
DCD	35 (7.6%)
DBD	426 (92.2%)
CMV risk status	
High (D+/R−)	65 (14.1%)
Intermediate (R+)	338 (73.2%)
Low (D−/R−)	59 (12.8%)

Abbreviations: BMI, body mass index; CCI, Charlson Comorbidity Index; DBD, donation after brain death; DCD, donation after cardiac death; HCC, hepatocellular carcinoma; MDRO, multi drug resistant organism; MELD, model end stage liver disease; NASH, nonalcoholic steatohepatitis; VRE, vancomycin resistant *Enterococcus* spp.

### 4.2 Antibiotic Exposures

During the 30 days preceding the transplant, 41.6% (192/462) of patients received at least one antibiotic, including daily SBP prophylaxis for 29.7% (137/462) (Table S6). Patients who received antibiotics prior to transplant were more likely to have been hospitalized in the month preceding the transplant (100% vs. 54%, *p* < 0.001) and have higher MELD scores (MELD > 21 in 68% of antibiotic‐exposed vs. 23% no antibiotic exposure, *p* < 0.001). The most frequent pretransplant antibiotic exposures were ceftriaxone (19%), followed by piperacillin–tazobactam (18%). A total of 58% (111/192) of pretransplant antibiotics were anaerobe‐targeting and were administered for a median cumulative duration of 5 days (IQR; 2, 9) compared to 10 days (IQR; 4, 19) for antibiotics without anaerobic coverage. During the 12 months following the transplant, approximately 82.9% (383/462) of patients received at least one antibiotic, with a median cumulative exposure of 13 days (IQR; 7, 24). The most frequent antibiotics prescribed were piperacillin–tazobactam (51.6%) and ceftriaxone (19.0%). A total of 65% (302/462) of antibiotics prescribed posttransplant were anaerobe‐targeting, administered for a median cumulative exposure of 7 days (IQR; 4, 14), while anaerobe‐sparing antibiotics were prescribed for 64.9% (300/462) for a median cumulative exposure of 11 days (IQR; 5, 21).

### 4.3 Pretransplant Antibiotic Exposure and Outcomes Following Transplant

**FIGURE 1:**
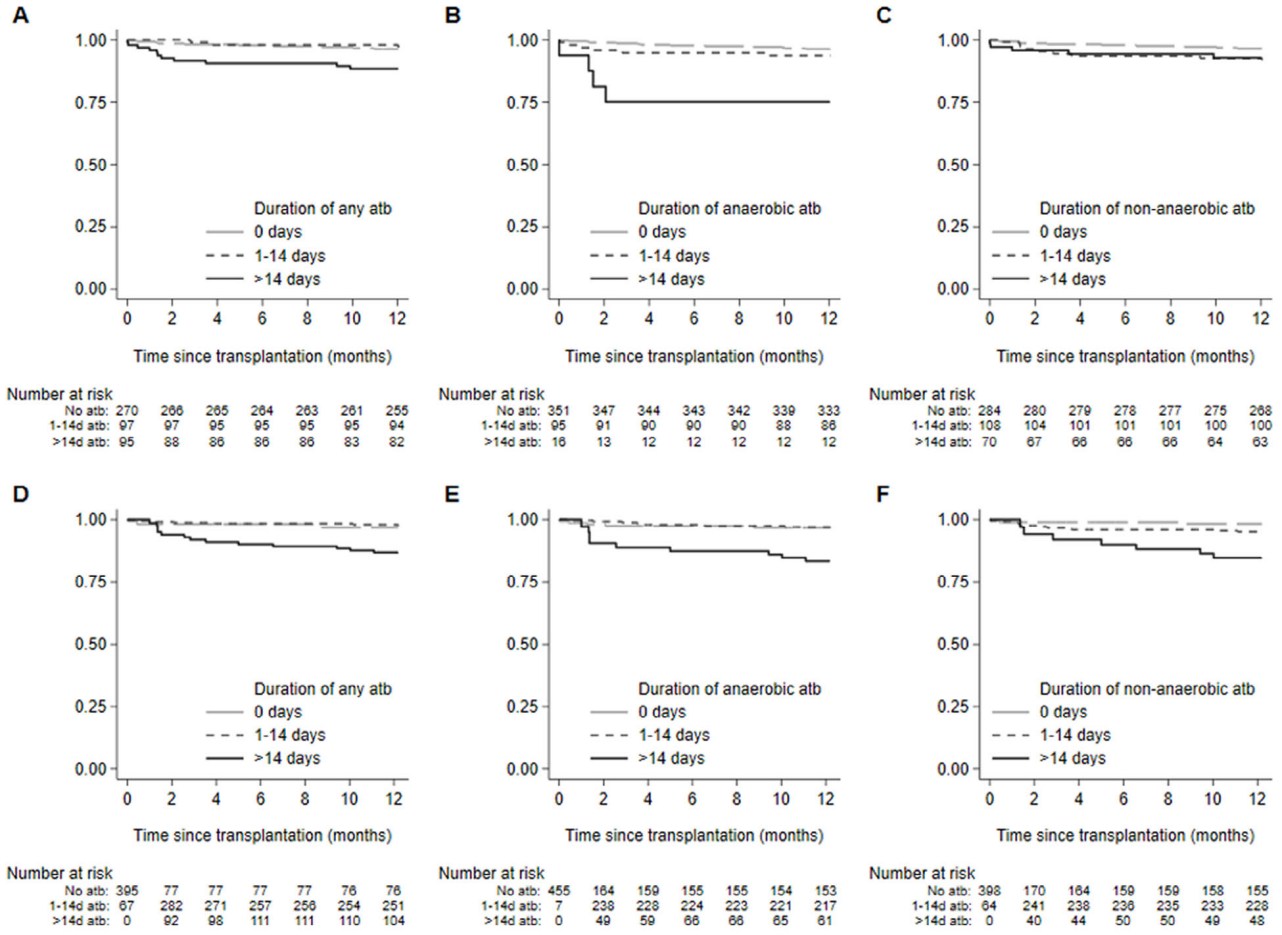
Kaplan–Meier of the duration of pretransplant (A) any antibiotic, (B) anaerobic and (C) non‐anaerobic antibiotics and posttransplant exposure to (D) any, (E) anaerobic, and (F) non‐anaerobic antibiotic up to 1‐year and 1‐year survival posttransplant.

The 1‐year survival after transplant was 95% (95% CI: 93%, 97%) with 24 deaths. We first explored the association between antibiotic exposure during the 30 days prior to transplant and 1‐year survival. In univariable analyses, exposure to an antibiotic of any spectrum or duration was associated with a significantly reduced 1‐year survival, with the strongest association observed with more than 14 days of anaerobic antibiotics (HR 7.40; 95% CI 2.43 to 22.49; *p* < 0.001) (Figure 1A–C, Table S7). In multivariable analyses, after adjustment for other pretransplant variables associated with 1‐year survival (Table S6), anaerobic antibiotics for longer than 14 days remained associated with worse survival at 1 year (HR 3.56; 95% CI 0.97–13.02; *p* = 0.055).

**FIGURE 2:**
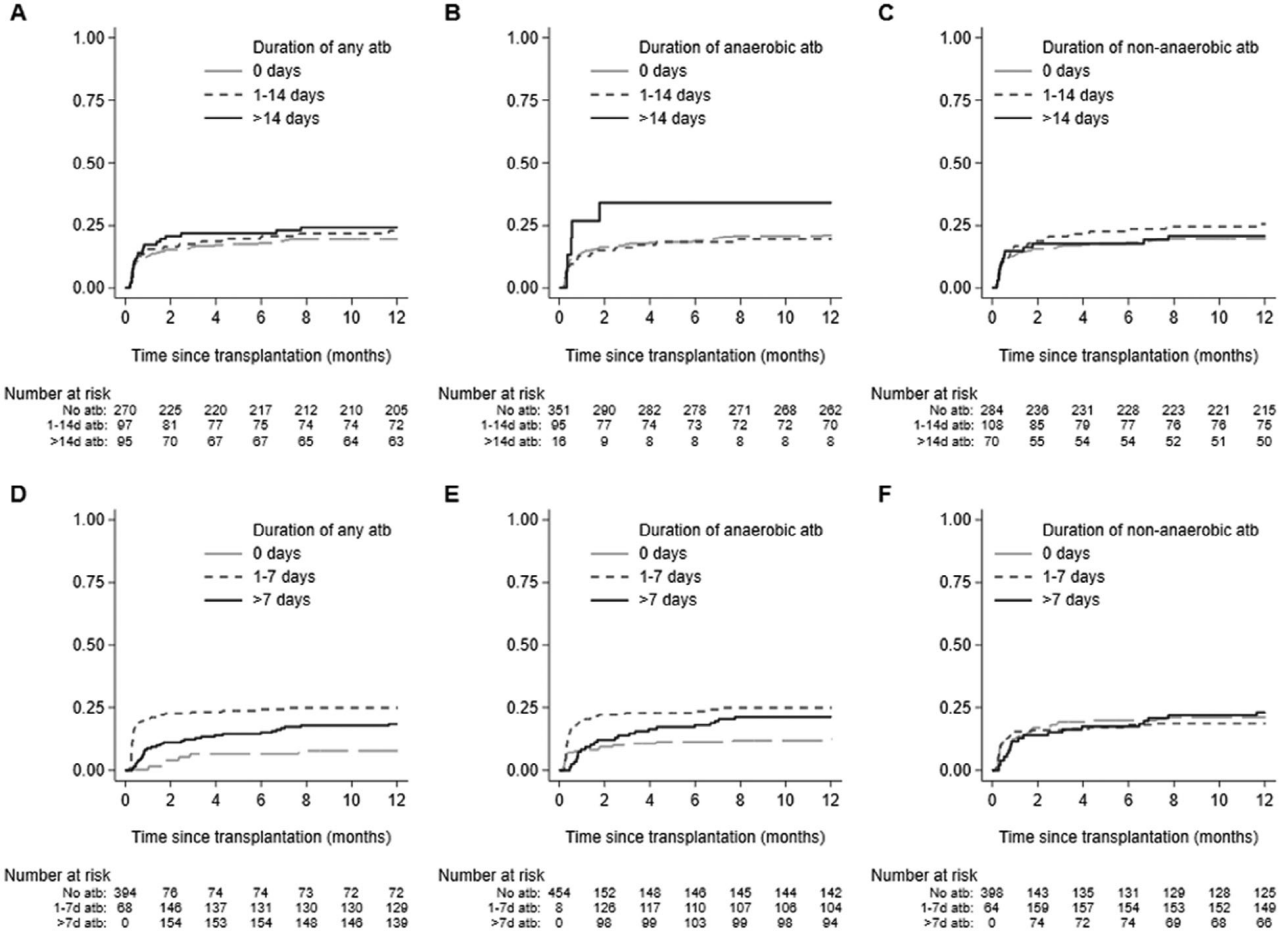
Kaplan‐Meier of the duration of pretransplant (A) any, (B) anaerobic, and (C) non‐anaerobic antibiotics and posttransplant exposure to (D) any, (E) anaerobic, and (F) non‐anaerobic antibiotics up to 1 year and 1‐year incidence of rejection during 1 year posttransplant.

No association between pretransplant antibiotics and the incidence of rejection following LTx was identified. (Figure 2A–C).

### 4.4 Posttransplant Antibiotic Exposure and Outcomes Following Transplant

A cumulative duration of more than 14 days of any spectrum of activity of antibiotics was significantly associated with reduced 1‐year survival in univariable analyses (Figure 1D–F). Following adjustment in multivariable models that included pretransplant anaerobic antibiotic exposure, we observed that more than 14 days of any antibiotic remained significantly associated with reduced survival (any antibiotic [HR 6.58; 95% CI 1.15–37.72; *p* = 0.035], anaerobic [HR 6.1; 95% CI 1.68–22.17; *p* = 0.006], non‐anaerobic [HR 7.07; 95% CI 1.53–32.71; *p* = 0.012]) (Table 2, Models 1–3). Among those who received more than 14 days of anaerobic antibiotics, the median (IQR) time to death from first antibiotic exposure was 50 days (IQR 20, 252). To further explore the influence of the antibiotic spectrum of activity on the association with 1‐year survival, we built a multivariable model that included both anaerobic and non‐anaerobic exposures following transplant (Table 2, Model 4). Following this adjustment, only exposure to anaerobic antibiotics, either before or after transplant, remained significantly associated with worse 1‐year survival (pretransplant [HR 4.31; 95% CI 1.17–15.29; *p* = 0.028) and posttransplant [HR 4.28; 95% CI 1.07–17.16; *p* = 0.040]), while exposure to non‐anaerobic antibiotics was not (HR 3.76; 95% CI 0.72–19.70; *p* = 0.117). In subsequent subgroup sensitivity analysis, with adjustment for tacrolimus trough concentrations, we observed similar associations between posttransplant antibiotic exposure and reduced OS at 1 year (Table S8). When we analyzed the effect of antibiotic exposure and 5‐year survival, we observed similar associations between pretransplant and posttransplant antibiotics.

**TABLE 2:** 1‐year survival after liver transplant: Multivariable analyses for pretransplant and posttransplant antibiotic exposure and the association of posttransplant exposure of > 14 days to any antibiotic are presented in Model 1, any anaerobic antibiotic in Model 2, any non‐anaerobic antibiotic in Model 3, and the concurrent effect of anaerobic and non‐anaerobic antibiotics in Model 4. All models are adjusted for pretransplant exposure of > 14 days to anaerobic antibiotics, liver transplant indication (autoimmune liver disease, other), VRE colonization, hemodialysis, MELD score, and posttransplant BSI (variables with *p* < 0.10 on univariable, Table S3).

		Model 1			Model 2			Model 3			Model 4		
		HR	95% CI	*p*	HR	95% CI	*p*	HR	95% CI	*p*	HR	95% CI	*p*
Post LTx any Abx	0 days	Ref											
	1–14 days	1.04	[0.19, 5.56]	0.968									
	> 14 days	6.58	[1.15, 37.72]	0.035									
Post LTx anaerobic Abx	0 days				Ref						Ref		
	1–14 days				1.37	[0.42, 4.53]	0.601				1.27	[0.38, 4.24]	0.696
	> 14 days				6.1	[1.68, 22.17]	0.006				4.28	[1.07, 17.16]	0.04
Post LTx non‐anaerobic Abx	0 days							Ref			Ref		
	1–14 days							2.2	[0.61, 7.85]	0.226	1.94	[0.53, 7.11]	0.316
	> 14 days							7.07	[1.53, 32.71]	0.012	3.76	[0.72, 19.70]	0.117
Pre LTx anaerobic Abx	0 days	Ref			Ref			Ref			Ref		
	1–14 days	0.57	[0.17, 1.94]	0.373	0.67	[0.21, 2.19]	0.51	0.64	[0.19, 2.15]	0.471	0.63	[0.19, 2.09]	0.45
	> 14 days	4.33	[1.20, 15.68]	0.026	4.37	[1.19, 16.02]	0.026	3.95	[1.08, 14.53]	0.038	4.31	[1.17, 15.92]	0.028
Autoimmune liver disease		3.56	[1.20, 10.54]	0.022	3.4	[1.14, 10.14]	0.028	3.13	[1.07, 9.13]	0.037	3.24	[1.10, 9.58]	0.033
Other liver disease		10.39	[1.29, 83.46]	0.028	9.43	[1.16, 76.95]	0.036	8.31	[1.01, 68.37]	0.049	6.49	[0.74, 56.83]	0.091
VRE colonized pretransplant		2.54	[1.05, 6.17]	0.039	2.48	[1.02, 6.04]	0.045	2.16	[0.88, 5.28]	0.093	2.33	[0.95, 5.69]	0.064
Hemodialysis pretransplant		1.42	[0.52, 3.86]	0.497	1.33	[0.47, 3.73]	0.592	1.51	[0.55, 4.12]	0.421	1.24	[0.44, 3.48]	0.684
MELD score		1	[0.95, 1.05]	0.989	1	[0.95, 1.05]	0.99	1	[0.95, 1.05]	0.933	1	[0.95, 1.05]	0.895
BSI posttransplant		1.78	[0.63, 5.07]	0.278	2.02	[0.70, 5.84]	0.196	1.86	[0.63, 5.53]	0.262	1.66	[0.56, 4.92]	0.36

Abbreviations. Abx, antibiotic; BSI, bloodstream infection; LTx, liver transplant; MELD, model end‐stage liver disease score; VRE, Vancomycin resistant Enterococcus.

Next, we explored antibiotic exposures during the posttransplant period and the incidence of rejection. We observed that rejection typically occurred early, on median Day 15 posttransplant (Table S3). Of the 102 recipients with an episode of rejection, 92.2% (94/102) had exposures to antibiotics exceeding routine perioperative prophylaxis preceding the rejection episode, the majority of which were anaerobe‐targeting 77.7% (73/94). Antibiotics were commenced on median Day 7 posttransplant (IQR; 1, 5), and 74.0% (54/73) were still receiving anaerobic antibiotics on the day of rejection confirmation. In univariate analyses, we observed that exposure to any antibiotic (HR 3.32; 95% CI 1.51–7.32; *p* = 0.003) or anaerobic antibiotic (HR 2.24; 95% CI 1.39–3.61; *p* = 0.001) was significantly associated with an increased incidence of subsequent rejection (Figure 2D–F, Table 3). In contrast, there was no association between only non‐anaerobic antibiotic exposures and risk of rejection (HR 1.0; 95% CI 0.66–1.50; *p* = 0.992). Longer durations of antibiotics did not significantly affect the negative impact antibiotic exposure had on increasing the risk of rejection compared to shorter durations. Following adjustment for other factors associated with 1‐year incidence of rejection (Table S5) in multivariable analyses, we observed that exposure to any antibiotic (HR 3.50; 95% CI 1.48–8.25; *p* = 0.004) or any anaerobic antibiotic (HR 2.34; 95% CI 1.43–3.84; *p* = 0.001) remained significantly associated with increased risk of rejection (Table 3). In subsequent subgroup sensitivity analysis, with adjustment for tacrolimus trough concentrations, we observed similar associations between posttransplant antibiotic exposure and incidence of rejection at 1 year (Table S9). When we analyzed the effect of antibiotic exposure and both the 3‐month incidence and the 5‐year incidence of rejection after transplant in sensitivity analyses, we observed similar associations between posttransplant anaerobic antibiotics and risk of rejection.

**TABLE 3:** 1‐year incidence of rejection after liver transplant: univariable and multivariable analyses for posttransplant antibiotic exposure and cumulative incidence of rejection during the first year after liver transplant with adjustment for Charlson Comorbidity Index, MELD score, biliary, HCV, and redo indication, cold ischemia time, and high‐risk CMV match status (*p* < 0.1 on univariable analysis, Table S4).

	Univariable			Multivariable analyses		
	HR	95% CI	*p* value	HR	95% CI	*p* value
Binary variable						
Any Abx	3.32	[1.51, 7.32]	0.003	3.5	[1.48, 8.25]	0.004
Any anaerobic Abx	2.24	[1.39, 3.61]	0.001	2.34	[1.43, 3.84]	0.001
Any non‐anaerobic Abx	1	[0.66, 1.50]	0.992	0.9	[0.58, 1.38]	0.624
Categories of duration						
Total Abx duration						
0 days	Ref			Ref		
1–7 days	3.81	[1.71, 8.49]	0.001	3.86	[1.62, 9.20]	0.002
> 7 days	2.66	[1.15, 6.16]	0.022	2.87	[1.15, 7.15]	0.023
Anaerobic Abx duration						
0 days	Ref			Ref			
1–7 days	2.22	[1.34, 3.67]	0.002	2.28	[1.36, 3.83]	0.002
> 7 days	2.3	[1.27, 4.16]	0.006	2.5	[1.34, 4.64]	0.004
Non‐anaerobic Abx duration						
0 days	Ref			Ref		
1–7 days	0.91	[0.58, 1.42]	0.676	0.82	[0.52, 1.30]	0.406
> 7 days	1.27	[0.72, 2.24]	0.403	1.15	[0.64, 2.07]	0.641

Abbreviations: Abx, antibiotic; CI, confidence interval, HR, hazard ratio.

## 5 Discussion

This is one of the first explorations of the potential effects of antibiotic exposure on key post‐solid organ transplant outcomes. We observed that more than 14 days of anaerobe‐targeting antibiotics either before or after transplant was associated with increased risk of death, while only posttransplant anaerobic antibiotic exposure was significantly associated with an increased incidence of rejection.

While novel in the setting of solid organ transplant, associations between broad‐spectrum antibiotics and adverse clinical outcomes have been observed in other immunocompromised patient cohorts [13, 14]. In hospitalized adults, anaerobic antibiotic exposure has been associated with reduced infection‐free and overall survival and increased risk of rehospitalization with sepsis in subsequent 90 days [15, 16, 17]. In the setting of cancer and immune compromise, exposure to broad‐spectrum anaerobe‐targeting antibiotics has been associated with reduced OS and PFS following CAR T‐cell therapy, increased risk of GVHD following allogeneic stem cell transplant, and reduced response to cancer immune checkpoint inhibitor therapy [14, 18, 19]. Building on these observations, we built robust models incorporating key transplant‐associated confounders, including surgical complications, infection, and immunosuppression, to explore the influence of the timing and duration of anaerobic antibiotic exposure on important LTx outcomes. We observed that the negative association between exposure to anaerobe‐targeting antibiotics and overall survival was not tied to the timing of exposure relative to transplant but rather the cumulative total exposure. In contrast, the impact of anaerobic antibiotics on the incidence of rejection was associated only with exposure that occurred following transplant. More prolonged durations of exposure were not associated with additional risk, and this is likely explained by the fact that the median time to rejection was Day 15 posttransplant, preventing recipients from experiencing more prolonged durations of antibiotic exposure prior to rejection diagnosis.

In healthy conditions, the microbial communities in the gut exert an important influence on host outcomes mediated through interaction with the immune system [20]. The gut microbiota is in symbiosis with the host, demonstrating stability and resilience in composition and diversity over time [21]. In humans, exposure to broad‐spectrum anaerobic antibiotics is associated with immediate and enduring shifts in the microbiota, including loss of healthy short‐chain fatty acid‐producing bacteria *Lachnospiraceae*, *Ruminococcaceae*, and *Faecalibacterium* and enrichment in antimicrobial resistant genes [22, 23]. The impacts of antibiotics on microbiota disruption are heterogeneous, with recovery shown to take several months, providing a biologically plausible mechanism through which antibiotic exposures may influence outcomes remote from their exposure [24, 25, 26]. There is a unique bidirectional relationship between the gut microbiota and the liver, established by the portal vein, which facilitates the passage of microbiota and microbiota‐derived products directly to the liver [27]. While ESLD is associated with a markedly disrupted microbiome characterized by loss of diversity and expansion of potential pathobionts, significant recovery of the microbiota has been observed following LTx [28, 29, 30, 31]. Persistent disruption of the microbiota post‐LTx has been associated with increased risk of subsequent rejection, non‐anastomotic biliary stricturing, bloodstream infection, and increased mortality [28, 30, 32]. We hypothesize that the interdependence between the gut microbiota and the liver may present a unique situation in the setting of LTx, where the allograft is particularly susceptible to the detrimental effects of antibiotic‐induced microbiota disruption. Future work will need to more precisely define the impact of antibiotic exposures on the microbiota of LTx recipients and define the mechanisms through which they influence the allograft and the host.

Our study was limited by its retrospective and single‐center design and may not necessarily reflect the broader experience. The retrospective nature of the study limited the variables we were able to capture to minimize confounding. For example, we did not include data on indications for antibiotic prescribing due to the pragmatic and subjective aspects of retrospectively quantifying and determining these data. Instead, we included objective surrogates of these variables, such as surgical complications, biopsy‐proven rejection, tacrolimus trough concentration, and microbiologically confirmed BSIs. The risk of residual confounding is difficult to quantify, and therefore these data should be replicated in larger, prospective cohorts. Nevertheless, our intention was to assess whether exposure to antibiotics in the peri‐transplant period might be associated with non‐infection‐related outcomes, potentially mediated by changes in the gut microbiome. We speculate that antibiotic‐induced dysbiosis decreases the diversity of the gut microbiota and negatively impacts the population of anaerobic commensals associated with immune tolerance. Replication of these findings in other cohorts and confirmation in prospective studies with comprehensive gut microbiota sampling and clinical data are needed to confirm these hypotheses.

In our single‐center cohort, we found that antibiotic exposures were an independent predictor of survival and rejection following LTx. We have expanded upon the experience of others and built more robust models that account for both the spectrum and duration of antibiotics and the timing of antibiotic exposure and are considered important transplant‐associated confounders. While antibiotics are a well‐established and essential aspect of care in the setting of transplant, maximizing their positive intended effects while limiting adverse microbiota disruption represents an ongoing clinical challenge. These results provide an impetus to further characterize the relationship between antibiotic use, microbiota disruption, and cellular immunity in liver transplantation.

## Author Contributions

**Olivia C Smibert**: conceptualization, investigation, writing – original draft, methodology, writing – review and editing, formal analysis, project administration. **Sara Vogrin**: Writing – original draft, methodology, formal analysis. **Marie Sinclair**: writing – review and editing. **Avik Majumdar**: writing – review and editing. **Mohamed Nasra**: writing – review and editing, data curation. **Dinesh Pandey**: methodology, software, data curation. **Hossein Jahanabadi**: methodology, software, data curation. **Jason A Trubiano**: conceptualization, investigation, writing – review and editing, supervision. **Kate A Markey**: supervision, writing – review and editing, writing – original draft, conceptualization. **Monica A Slavin**: conceptualization, writing – review and editing, supervision. **Adam Testro**: writing – review and editing. **Jason C Kwong**: conceptualization, writing – review and editing, supervision.

## Conflicts of Interest

The authors of this manuscript have conflicts of interest to disclose. Kate A. Markey reports that she holds equity and is on the advisory board for Postbiotics Plus and consults for Crestone.

## Supporting information



Supporting Information

Visual Abstract

## Data Availability

De‐identified data that support the findings of this study are available for ethics‐approved studies from the corresponding author upon reasonable request. A data transfer agreement will be required.
