# Supplementary material for: Antibiotic Exposure and Risk of Allograft Rejection and Survival After Liver Transplant: An Observational Cohort Study From a Tertiary Referral Centre
Source: Transpl Infect Dis. 2025 Mar 28;27(3):e70026. doi: 10.1111/tid.70026 (PMC12205275; doi:10.1111/tid.70026)
Supplement: Supplementary file 1 — Supporting Information [file TID-27-e70026-s001.docx]

**Supplementary Data**

**Definitions**

Indications for liver transplant were classified as one of the following categories: Hepatitis B or C virus, autoimmune, non-alcoholic fatty liver disease (NASH), re-transplant for graft failure of any aetiology, alcohol-related liver disease, hepatoma, biliary (including primary and secondary sclerosing cholangitis, primary biliary cholangitis, biliary cirrhosis and biliary atresia) or other. For the transplant procedure and donor characteristics, cold ischemia time, donor type [either brain death (DBD) or cardiac death (DCD)], utilisation of OrganOx metra® perfusion system, donor and recipient ABO blood type and gender matched status were recorded. CMV risk status was determined as either high, intermediate or low risk based on donor and recipient CMV serology per standard criteria ^1^.

**Primary Exposure Variable**

Specific indications for antibiotic prescribing were not routinely documented in the electronic medical record. Antibiotics were classified as prophylactic if they were prescribed per the local protocol outlined in the centre specific protocols and were excluded from the primary analysis. All antibiotics that were not prescribed as prophylactic per institutional protocol were considered treatment related. Additional sensitivity analyses were undertaken that included all antibiotics.

**Statistical methods**

Descriptive statistics were used to examine demographic characteristics. Data were presented as means and SDs, medians and IQRs, or counts and percentages as appropriate. Categorical variables were compared using the Fisher’s exact test and continuous variables using the Mann-Whitney U Test. Missing demographic variables were grouped into a separate category “unknown.” If the patient was lost to follow-up, they were censored at their last visit at the health service (inpatient or outpatient). We acknowledge that death might be a competing risk when analysing time to rejection, however as time-varying covariates can not be interpreted in subdistribution hazard models, we have counted death as a censoring event. Incidence of death was low in the first year (4%) and occurred later than rejection (median time to rejection was 14 days, while median time to death was 52 days). We performed sensitivity analysis using competing risk regression according to method of Fine and Grey for the models including only pre-transplant variables and results were comparable to Cox regression analysis. Similarly cumulative incidence of rejection is almost identical for both methods. Additional sensitivity analysis included 5-year survival and 3-month rejection

**Table 1.** Summary of statistical models undertaken

| Main exposure of interest | | | **1-year survival** | **1-year incidence of rejection** |
| --- | --- | --- | --- | --- |
| Pre transplant antibiotic use (30-day prior tx) | Binary exposure | Any abx | Unadjusted  Adjusted for other baseline variables (p<0.1 on univariable: MELD, autoimmune indication, other indication, VRE colonisation, Haemodialysis) (**Supp table 6**) | Unadjusted  Adjusted for other baseline variables (p<0.1 on univariable: (CCI, biliary, HCV, Re-do indication, CMV status, Time cold, MELD (**not shown**) |
|  |  | Anaerobic abx |  |  |
|  |  | Non-anaerobic atb |  |  |
|  | DOT (continuous) | Any atb |  |  |
|  |  | Anaerobic abx |  |  |
|  |  | Non-anaerobic atb |  |  |
|  | DOT (categorical) | Any atb  (0, 1-14, >14 days) |  |  |
|  |  | Anaerobic abx  (0, 1-14, >14 days) |  |  |
|  |  | Non-anaerobic abx  (0, 1-14, >14 days) |  |  |
| Post transplant antibiotic use (1 years post tx), entered as time varying covariate | Binary exposure | Any abx | Unadjusted  Adjusted for other baseline variables (p<0.1 on univariable: autoimmune liver disease, other liver disease, VRE colonisation, heamodialyziz, MELD) &  Pre tx anaerobic abx & BSI post tx (as time varying covariate)  (**Table 2**)  *Subgroup analysis of only patients who received tacrolimus, same model but adjusted for tacrolimus level as well (****Supp Table 7****)* | Unadjusted  Adjusted for other baseline variables (p<0.1 on univariable: CCI, biliary, HCV, Re-do indication, CMV status, Time cold, MELD) (**Table 3**)  *Subgroup analysis of only patients who received tacrolimus, same model but adjusted for tacrolimus level as well (****Supp Table 8****)* |
|  |  | Anaerobic abx |  |  |
|  |  | Non-anaerobic abx |  |  |
|  | DOT (categorical) | Any abx  (0, 1-14, >14 days) |  | N/A due to the low numbers of patients with >7 days of antibiotic prior rejection |
|  |  | Anaerobic abx  (0, 1-14, >14 days) |  |  |
|  |  | Non-anaerobic abx  (0, 1-14, >14 days) |  |  |
|  | DOT (categorical) | Any abx  (0, 1-7, >7 days) | N/A | Unadjusted  Adjusted for other baseline variables (p<0.1 on univariable: CCI, biliary, HCV, Re-do indication, CMV status, Time cold, MELD) (**Table 3**)  *Subgroup analysis of only patients who received tacrolimus, same model but adjusted for tacrolimus level as well (****Supp Table 8****)* |
|  |  | Anaerobic abx  (0, 1-7 >7 days) |  |  |
|  |  | Non-anaerobic abx  (0, 1-7, >7 days) |  |  |

Sensitivity analysis (results not shown in the paper: same analysis using outcome of 5-year survival, 3-month rejection and 5-year rejection

**Table 2.** Characteristics of 462 deceased liver donors

| **Donor and Transplant Characteristics** | **N = 462** |
| --- | --- |
| Donor age, median (IQR) | 47 (32, 59) |
| Donor BMI, median (IQR) | 26 (23, 29) |
| Donor-Recipient match status  ABO blood group match  Gender matched | 375 (81.2%)  294 (64.1%) |
| Cold ischemia time  Median (IQR)  <12 hours  >12 hours | 376 (316, 456)  431 (93.3%)  31 (6.7%) |
| OrganOx metra® ex-vivo perfusion | 17 (3.7%) |
| Split Liver transplant | 34 (7.4%) |

IQR, interquartile range; BMI; body mass index

**Table 3.** Outcomes after transplant for 462 recipients after liver transplant

| **Transplant outcomes** | **N = 462** |
| --- | --- |
| Rejection  Any rejection after transplant  Rejection within 90 days of transplant  Rejection day, median (IQR) | 102 (22.1%)  81 (17.5%)  15 (9, 53) |
| Rejection diagnostic method  Histological on liver biopsy | 102 (100%) |
| Rejection Grade (RAI criteria)  Mild  Moderate  Severe | 39 (38.2%)  50 (49.0%)  13 (12.7%) |
| Surgical complications  Biliary  Intra-abdominal hematoma  Hepatic artery or portal vein thrombosis  Other | 41 (8.9%)  10 (24.4%)  16 (39.0%)  9 (22.0%)  6 (14.6%) |
| Blood stream infection (BSI)  BSI day, median (IQR)  Polymicrobial  Gram positive organism  Gram negative organism  Fungal organism | 48 (10.3%)  5 (1,21)  7 (14.6%)  27 (56.3%)  19 (39.6%)  14 (29.2%) |
| Hospital length of stay (days), median (IQR) | 16 (11, 27) |
| Allograft complications  Primary non function  Re-transplant  Re transplant day, median (IQR) | 16 (3.5%)  30 (6.5%)  16 (11, 27) |
| Death after transplant  Died  Day of death, median (IQR)  Death within 90 days of transplant  Death within 1 year of transplant | 63 (13.6%)  495 (110,1144)  14 (3.0%)  24 (5.2%) |
| Primary cause of Death at 1 year  Infection  Malignancy  Cardiovascular  Chronic allograft rejection  Multiorgan failure  Respiratory failure  Other | 1 (4.2%)  5 (20.8%)  6 (25.0%)  1 (4.2%)  4 (16.7%)  2 (8.3%)  5 (20.8%) |

**Table 4.** Univariable analyses of factors associated with 1-year survival following transplant

| Variable | Level | HR | 95% CI | P value |
| --- | --- | --- | --- | --- |
| Age |  | 1 | [0.97,1.04] | 0.859 |
| Sex |  | 1.15 | [0.49,2.68] | 0.749 |
| Days on transplant waitlist |  | 1 | [1.00,1.00] | 0.154 |
| BMI | BMI <25 | 1 | [1.00,1.00] |  |
|  | BMI 25-30 | 0.72 | [0.24,2.15] | 0.554 |
|  | BMI >30 | 1.03 | [0.40,2.66] | 0.957 |
|  | Unknown BMI | 1.37 | [0.30,6.32] | 0.690 |
| CCI | 0-2 points | 1 | [1.00,1.00] |  |
|  | 3-5 points | 1.76 | [0.38,8.15] | 0.469 |
|  | 6+ point | 2.09 | [0.47,9.25] | 0.332 |
|  | Unknown | 0 | [0.00,0.00] |  |
| Indication | Autoimmune | 4.15 | [1.55,11.11] | **0.005** |
|  | Biliary | 0.32 | [0.04,2.37] | 0.264 |
|  | ETOH | 0.62 | [0.18,2.07] | 0.434 |
|  | HBV | n/a |  |  |
|  | HCV | 0.93 | [0.35,2.49] | 0.885 |
|  | NASH | 0.69 | [0.16,2.94] | 0.618 |
|  | Other | 1.85 | [0.73,4.66] | 0.192 |
|  | Redo | 1.1 | [0.15,8.15] | 0.925 |
| Hepatocellular carcinoma |  | 0.64 | [0.22,1.89] | 0.420 |
| MELD score (categorical) | 0-1 | Ref |  |  |
|  | 2 | 1.93 | [0.59,6.32] | 0.278 |
|  | 3 | 2.3 | [0.82,6.44] | 0.114 |
| MELD score (continuous) |  | 1.04 | [1.00,1.08] | **0.057** |
| VRE colonised |  | 2.72 | [1.22,6.07] | **0.015** |
| MDRO colonised |  | 1.25 | [0.43,3.67] | 0.681 |
| Medications | PPI | 1.88 | [0.84,4.18] | 0.122 |
|  | Lactulose | 1.71 | [0.77,3.80] | 0.191 |
|  | Rifaximin | 1.04 | [0.45,2.44] | 0.921 |
| During the 30 days preceding transplant | Hospitalisation | 2.60 | [0.78,8.73] | 0.121 |
|  | Admit to ICU | 2.10 | [0.83,5.28] | 0.116 |
|  | Haemodialysis | 3.36 | [1.33,8.47] | **0.010** |
|  | Mechanical ventilation | 1.96 | [0.88,4.37] | 0.101 |
| Donor | DCD vs DBD | 0.53 | [0.07,3.90] | 0.529 |
|  | ABO match | 0.69 | [0.27,1.74] | 0.434 |
|  | OrganOx perfusion | 2.56 | [0.60,10.90] | 0.203 |
|  | Cold ischemia time | 1.00 | [1.00,1.00] | 0.133 |
|  | Gender match | 1.70 | [0.68,4.29] | 0.258 |
| CMV status | High | 0.49 | [0.11,2.09] | 0.334 |
|  | Intermediate | Ref |  |  |
|  | Low | 0.27 | [0.04,1.98] | 0.197 |
| Surgical Complication |  | 2.15 | [0.73, 6.28] | 0.163 |
| Blood Stream Infection |  | 2.24 | [1.19, 4.19] | **0.012** |

BMI, body mass index; CCI, Charlson Comorbidity Index; MELD score, model end-stage liver disease score; VRE, vancomycin resistant enterococcus; MDRO, multidrug resistant organism; HR, hazard ratio; CI, confidence interval

**Table 5.** Univariable analyses of factors associated with 1-year incidence of rejection following transplant

| Variable |  | Level | HR | 95% CI | p value |
| --- | --- | --- | --- | --- | --- |
| Age |  |  | 0.99 | [0.97,1.00] | 0.152 |
| Sex |  |  | 0.76 | [0.51,1.14] | 0.182 |
| Days on transplant waitlist |  |  | 1 | [1.00,1.00] | 0.418 |
| BMI |  | BMI <25 | Ref |  |  |
|  |  | BMI 25-30 | 1.15 | [0.70,1.89] | 0.570 |
|  |  | BMI >30 | 0.95 | [0.58,1.57] | 0.847 |
|  |  | Unknown BMI | 0.91 | [0.36,2.32] | 0.841 |
| CCI |  | 0-2 points | Ref |  |  |
|  |  | 3-5 points | 0.58 | [0.33,0.99] | **0.047** |
|  |  | 6+ point | 0.56 | [0.33,0.94] | **0.029** |
|  |  | Unknown | 0.39 | [0.05,2.87] | 0.353 |
| Indication |  | Autoimmune | 1.68 | [0.85,3.34] | 0.139 |
|  |  | Biliary | 1.70 | [1.00,2.87] | **0.048** |
|  |  | ETOH | 0.91 | [0.54,1.54] | 0.725 |
|  |  | HBV | 0.71 | [0.33,1.53] | 0.384 |
|  |  | HCV | 0.44 | [0.23,0.82] | **0.010** |
|  |  | NASH | 0.68 | [0.33,1.40] | 0.291 |
|  |  | Other | 1.11 | [0.65,1.90] | 0.696 |
|  |  | Redo | 3.68 | [1.91,7.09] | **<0.001** |
| Hepatocellular carcinoma |  |  | 0.66 | [0.39,1.11] | 0.118 |
| MELD score (categorical) |  | 0-1 | Ref |  |  |
|  |  | 2 | 1.01 | [0.59,1.75] | 0.961 |
|  |  | 3 | 1.12 | [0.71,1.76] | 0.638 |
| MELD score (continuous) |  |  | 1.02 | [1.00,1.05] | **0.025** |
| VRE colonised |  |  | 0.98 | [0.63,1.51] | 0.913 |
| MDRO colonised |  |  | 0.7 | [0.36,1.35] | 0.286 |
| Medications |  | PPI | 1.06 | [0.70,1.60] | 0.788 |
|  |  | Lactulose | 1.28 | [0.86,1.92] | 0.227 |
|  |  | Rifaxamin | 1.40 | [0.93,2.10] | 0.110 |
| During the 30 days preceding transplant |  | Hospitalisation | 1.10 | [0.69,1.75] | 0.679 |
|  |  | Admit to ICU | 1.40 | [0.83,2.37] | 0.205 |
|  |  | Haemodialyses | 1.61 | [0.90,2.90] | 0.110 |
|  |  | Mechanical ventilation | 1.11 | [0.74,1.68] | 0.603 |
| Donor |  | DCD vs DBD | 1.28 | [0.64,2.54] | 0.481 |
|  |  | ABO match | 0.77 | [0.48,1.24] | 0.276 |
|  |  | OrganOx perfusion | 0.54 | [0.13,2.19] | 0.388 |
|  |  | Cold ischaemia time | 1 | [1.00,1.00] | **0.090** |
|  |  | Gender match | 0.92 | [0.60,1.39] | 0.676 |
| CMV status |  | High | 1.90 | [1.15,3.11] | **0.012** |
|  |  | Intermediate | Ref |  |  |
|  |  | Low | 1.35 | [0.75,2.41] | 0.314 |
| Surgical Complication |  |  | 0.79 | [0.37, 1.65] | 0.525 |
| Blood Stream Infection |  |  | 0.86 | [0.43, 1.71] | 0.664 |

HR, hazard ratio; CI, confidence interval; BMI, body mass index; CCI, Charlson Comorbidity Index; MELD score, model end-stage liver disease score; VRE, vancomycin resistant enterococcus; MDRO, multidrug resistant organism

**Table 6.** Antibiotic utilisation during 30 days prior to and for 12 months following transplant

| **Antibiotic utilisation pre transplant** | **N = 462** |
| --- | --- |
| Spontaneous bacterial peritonitis (SBP) prophylaxis | 137 (29.7%) |
| Any antibiotic  Antibiotic days of those with antibiotic, median (IQR) | 192 (41.6%)  13.5 (5, 24) |
| Any anaerobic antibiotic  Duration of anaerobic antibiotic in those who received, median (IQR) | 111 (24.0%)  6 (3, 10) |
| Any non-anaerobic antibiotic  Duration of non-anaerobic antibiotic in those who received, median (IQR) | 178 (38.5%)  10 (4, 19) |
| **Antibiotic utilisation post-transplant** | |
| *Pneumocystis jirovecii* pneumonia (PJP) Prophylaxis | 462 (100%) |
| Any antibiotic exposure after exclusion of prophylaxis during 1 year post LTx  Antibiotic days of those with antibiotic, median (IQR) | 383 (82.9%)  13 (7,24) |
| Any anaerobic antibiotics during 1 year post LTx  Duration of anaerobic antibiotic in those who recieved, median (IQR) | 302 (65.4%)  7 (4, 14) |
| Any non-anaerobic antibiotic during 1 year post LTx  Duration of non-anaerobic antibiotic in those who recieved, median (IQR) | 421 (91.1%)  11 (5, 21) |

LTx, Liver transplant

**Table 7.** 1-year survival after liver transplant: Uni and multivariable analyses for antibiotic exposure pre-transplant and 1-year survival after liver transplant. Multivariable analyses adjusted for MELD score, Autoimmune indication, Other indication, VRE colonisation and haemodialysis pre transplant (variables with p<0.1 on univariable analysis, Supplementary Table 3))

|  | | Univariable analyses | | | Multivariable analyses | | |
| --- | --- | --- | --- | --- | --- | --- | --- |
|  | | HR | 95% CI | P value | HR | 95% CI | P value |
| **Binary variable** | | | | | | | |
| Any Abx | | 2.01 | [0.89,4.53] | 0.092 | 0.99 | [0.35,2.81] | 0.992 |
| Any anaerobic Abx | | 2.36 | [1.05,5.32] | 0.038 | 1.29 | [0.47,3.57] | 0.619 |
| Any non-anaerobic Abx | | 2.29 | [1.02,5.15] | 0.046 | 1.2 | [0.43,3.34] | 0.726 |
| **Continuous variable** | | | | | | | |
| Any Abx | | 1.03 | [1.00,1.06] | 0.023 | 1.01 | [0.97,1.04] | 0.745 |
| Anaerobic Abx | | 1.1 | [1.04,1.15] | <0.001 | 1.07 | [1.01,1.14] | 0.032 |
| Non-anaerobic Abx | | 1.02 | [0.98,1.06] | 0.274 | 0.98 | [0.94,1.03] | 0.489 |
| **Categories of duration** | | | | | | | |
| Any Abx (days) | |  |  |  |  |  |  |
|  | 0 days | Ref |  |  | Ref |  |  |
|  | 1-14 days | 0.83 | [0.23,3.00] | 0.773 | 0.61 | [0.15,2.43] | 0.481 |
|  | >14 days | 3.30 | [1.40,7.77] | 0.006 | 1.44 | [0.46,4.54] | 0.530 |
| Anaerobic Abx (days) | |  |  |  |  |  |  |
|  | 0 days | Ref |  |  | Ref |  |  |
|  | 1-14 days | 1.63 | [0.62,4.23] | 0.32 | 0.93 | [0.30,2.88] | 0.900 |
|  | >14 days | 7.40 | [2.43,22.49] | <0.001 | 3.56 | [0.97,13.02] | 0.055 |
| Non-anaerobic Abx (days) | |  |  |  |  |  |  |
|  | 0 days | Ref |  |  | Ref |  |  |
|  | 1-14 days | 2.42 | [0.98,5.94] | 0.055 | 1.42 | [0.49,4.13] | 0.524 |
|  | >14 days | 2.09 | [0.71,6.12] | 0.178 | 0.88 | [0.25,3.17] | 0.849 |

HR, hazard ratio; CI, confidence interval; Abx, antibiotic

**Table 8**. 1-year survival after liver transplant: Uni and multivariable analyses for antibiotic exposure post-transplant and 1-year survival after liver transplant in subset of 438 subjects receiving Tacrolimus immunosuppression. Multivariable analyses (A) adjusted for MELD score, Autoimmune indication, VRE colonisation, haemodialysis pre transplant (variables with p<0.1 on univariable analysis, Supplementary Table 3) and multivariable analysis (B) additionally adjusted for Tacrolimus trough levels.

|  | Unadjusted | | | Adjusted as in primary analysis (A) | | | Adjusted also for Tacrolimus trough levels (B) | | |
| --- | --- | --- | --- | --- | --- | --- | --- | --- | --- |
|  | HR | 95% CI | p | HR | 95% CI | p | HR | 95% CI | p |
| **Binary variable** | | | | | | | | | |
| Any Abx | 1.83 | [0.42,7.93] | 0.419 | 1.04 | [0.23,4.76] | 0.959 | 1.09 | [0.24,4.98] | 0.911 |
| Any anaerobic Abx | 2.4 | [0.79,7.26] | 0.121 | 1.88 | [0.60,5.87] | 0.277 | 1.82 | [0.58,5.66] | 0.303 |
| Any non-anaerobic Abx | 5.09 | [1.17,22.03] | 0.03 | 3.12 | [0.68,14.38] | 0.145 | 3.09 | [0.67,14.25] | 0.147 |
| **Categories of duration** | | | | | | | | | |
| Total Abx duration |  |  |  |  |  |  |  |  |  |
| 0 days | Ref |  |  | Ref |  |  | Ref |  |  |
| 1-14 days | 0.72 | [0.14,3.72] | 0.697 | 0.48 | [0.09,2.55] | 0.386 | 0.49 | [0.09,2.64] | 0.408 |
| >14 days | 5.68 | [1.23,26.24] | 0.026 | 3.47 | [0.69,17.46] | 0.131 | 3.56 | [0.71,17.78] | 0.121 |
| Anaerobic Abx duration |  |  |  |  |  |  |  |  |  |
| 0 days | Ref |  |  | Ref |  |  | Ref |  |  |
| 1-14 days | 1.21 | [0.34,4.28] | 0.772 | 1.1 | [0.31,3.93] | 0.889 | 1.06 | [1.48,4.21] | 0.001 |
| >14 days | 8.06 | [2.40,27.00] | 0.001 | 6.14 | [1.65,22.79] | 0.007 | 5.69 | [2.26,8.81] | 0 |
| Non-anaerobic Abx duration |  |  |  |  |  |  |  |  |  |
| 0 days | Ref |  |  | Ref |  |  | Ref |  |  |
| 1-14 days | 3.49 | [0.77,15.96] | 0.106 | 2.46 | [0.52,11.71] | 0.259 | 2.43 | [0.59,1.59] | 0.895 |
| >14 days | 15.59 | [3.19,76.20] | 0.001 | 9.58 | [1.70,54.08] | 0.011 | 9.95 | [0.89,3.38] | 0.103 |

HR, hazard ratio; CI, confidence interval; Abx, antibiotic

**Table 9**. 1-year incidence of rejection after liver transplant: Univariable and multivariable analyses for post-transplant antibiotic exposure and cumulative incidence of rejection during the first year after liver transplant in subset of 438 subjects receiving Tacrolimus immunosuppression. Multivariable analyses (A) adjusted for Charlson Comorbidity Index, MELD score, Biliary, HCV and Redo indication, Cold Ischaemia time and High risk CMV match status (p<0.1 on univariable analysis, Supplementary Table 4) as in primary analysis and multivariable analysis (B) additionally adjusted for Tacrolimus trough level.

|  | Unadjusted | | | Adjusted as in primary analysis (A) | | | Adjusted also for Tacrolimus trough levels (B) | | |
| --- | --- | --- | --- | --- | --- | --- | --- | --- | --- |
|  | HR | 95% CI | p | HR | 95% CI | p | HR | 95% CI | p |
| **Binary variable** | | | | | | | | | |
| Any Abx | 2.31 | [1.22,4.36] | 0.01 | 2.41 | [1.25,4.63] | 0.009 | 2.5 | [1.30,4.80] | 0.006 |
| Any anaerobic Abx | 2.5 | [1.59,3.95] | 0 | 2.64 | [1.62,4.31] | 0 | 2.85 | [1.74,4.66] | 0 |
| Any non-anaerobic Abx | 1.11 | [0.72,1.72] | 0.636 | 1.1 | [0.69,1.73] | 0.693 | 1.09 | [0.69,1.73] | 0.717 |
| **Categories of duration** | | | | | | | | | |
| Total Abx duration |  |  |  |  |  |  |  |  |  |
| 0 days | Ref |  |  | Ref |  |  | Ref |  |  |
| 1-7 days | 2.16 | [1.13,4.15] | 0.021 | 2.28 | [1.18,4.42] | 0.014 | 2.37 | [1.22,4.59] | 0.01 |
| >7 days | 2.7 | [1.32,5.54] | 0.007 | 2.81 | [1.29,6.10] | 0.009 | 2.93 | [1.34,6.40] | 0.007 |
| Anaerobic Abx duration |  |  |  |  |  |  |  |  |  |
| 0 days | Ref |  |  | Ref |  |  | Ref |  |  |
| 1-7 days | 2.16 | [1.32,3.55] | 0.002 | 2.28 | [1.36,3.82] | 0.002 | 2.49 | [1.48,4.21] | 0.001 |
| >7 days | 3.82 | [2.09,6.97] | 0 | 4.36 | [2.23,8.50] | 0 | 4.46 | [2.26,8.81] | 0 |
| Non-anaerobic Abx duration |  |  |  |  |  |  |  |  |  |
| 0 days | Ref |  |  | Ref |  |  | Ref |  |  |
| 1-7 days | 0.99 | [0.62,1.58] | 0.964 | 0.98 | [0.60,1.59] | 0.931 | 0.97 | [0.59,1.59] | 0.895 |
| >7 days | 1.72 | [0.91,3.25] | 0.095 | 1.69 | [0.87,3.29] | 0.121 | 1.74 | [0.89,3.38] | 0.103 |

HR, hazard ratio; CI, confidence interval; Abx, antibiotic

References

1. Kotton CN, Kumar D, Caliendo AM, et al. Updated international consensus guidelines on the management of cytomegalovirus in solid-organ transplantation. *Transplantation*. Aug 27 2013;96(4):333-60. doi:10.1097/TP.0b013e31829df29d

2. Rodríguez-Perálvarez M, Colmenero J, González A, et al. Cumulative exposure to tacrolimus and incidence of cancer after liver transplantation. *American journal of transplantation : official journal of the American Society of Transplantation and the American Society of Transplant Surgeons*. Jun 2022;22(6):1671-1682. doi:10.1111/ajt.17021

3. Rodríguez-Perálvarez M, Guerrero M, De Luca L, et al. Area Under Trough Concentrations of Tacrolimus as a Predictor of Progressive Renal Impairment After Liver Transplantation. *Transplantation*. Dec 2019;103(12):2539-2548. doi:10.1097/tp.0000000000002760

4. Neuberger JM, Bechstein WO, Kuypers DR, et al. Practical Recommendations for Long-term Management of Modifiable Risks in Kidney and Liver Transplant Recipients: A Guidance Report and Clinical Checklist by the Consensus on Managing Modifiable Risk in Transplantation (COMMIT) Group. *Transplantation*. Apr 2017;101(4S Suppl 2):S1-s56. doi:10.1097/tp.0000000000001651
