# Supplementary material for: Antibiotic Exposure and Risk of Allograft Rejection and Survival After Liver Transplant: An Observational Cohort Study From a Tertiary Referral Centre
Source: Transpl Infect Dis. 2025 Mar 28;27(3):e70026. doi: 10.1111/tid.70026 (PMC12205275; doi:10.1111/tid.70026)
Supplement: Supplementary file 2 — Visual Abstract [file TID-27-e70026-s002.pptx]

## Slide 1
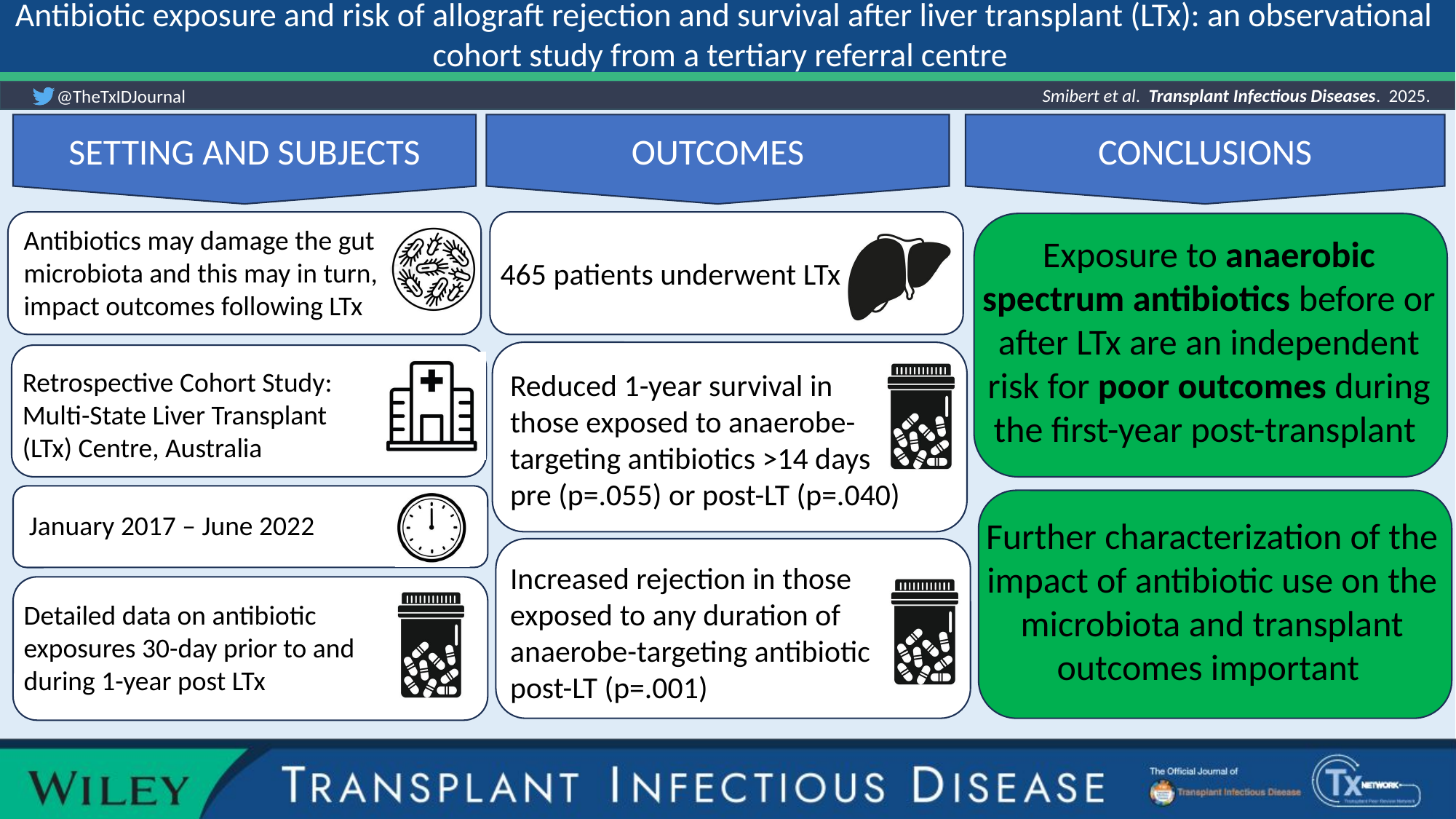

Antibiotic exposure and risk of allograft rejection and survival after liver transplant (LTx): an observational cohort study from a tertiary referral centre
Smibert et al. Transplant Infectious Diseases. 2025.
 @TheTxIDJournal
SETTING AND SUBJECTS
OUTCOMES
CONCLUSIONS
Antibiotics may damage the gut microbiota and this may in turn, impact outcomes following LTx
Exposure to anaerobic spectrum antibiotics before or after LTx are an independent risk for poor outcomes during the first-year post-transplant
465 patients underwent LTx
Retrospective Cohort Study: Multi-State Liver Transplant (LTx) Centre, Australia
Reduced 1-year survival in those exposed to anaerobe-targeting antibiotics >14 days pre (p=.055) or post-LT (p=.040)
January 2017 – June 2022
Further characterization of the impact of antibiotic use on the microbiota and transplant outcomes important
Increased rejection in those exposed to any duration of anaerobe-targeting antibiotic post-LT (p=.001)
Detailed data on antibiotic exposures 30-day prior to and during 1-year post LTx
